# Impact of autoantibodies against myelin oligodendrocyte glycoprotein in paediatric acquired demyelinating disease: Intellectual functioning and academic performance

**DOI:** 10.1016/j.ejpn.2024.09.001

**Published:** 2024-09-03

**Authors:** Daniel Griffiths-King, Charly Billaud, Lydiah Makusha, Ling Lynette Looi, Evangeline Wassmer, Sukhvir Wright, Amanda G. Wood

**Affiliations:** a Institute of Health and Neurodevelopment, College of Health and Life Sciences, Aston University, Birmingham, UK; b Nanyang Technological University, Singapore, Singapore; c Department of Neurology, Birmingham Children’s Hospital, Birmingham Women’s and Children’s Hospital NHS Foundation Trust, Birmingham, UK; d School of Psychology, Faculty of Health, Melbourne Burwood Campus, Deakin University, Geelong, Victoria, Australia

**Keywords:** MOGAD, Pediatric, Acquired demyelinating syndromes, Myelin oligodendrocyte glycoprotein autoantibodies, Intellectual functioning, Outcomes

## Abstract

Paediatric acquired demyelinating syndromes (pADS) attack white matter pathways in the brain during an important period of development. Affected children can experience poor functional outcomes, including deficits in specific cognitive domains. Understanding risk factors for poor outcome will guide clinical management of these children. One clinical phenotype which may differentially impact cognitive outcomes is the presence of autoantibodies to myelin oligodendrocyte glycoprotein (MOG). Preliminary research has suggested that cognitive difficulties exist in paediatric patients who test positive for MOG antibodies or MOGAD (Myelin Oligodendrocyte Glycoprotein Associated Disease) however, they experience a less severe profile compared to seronegative counterparts. The current study assesses children diagnosed with pADS who tested positive or negative for MOG-ab using standardised assessments of both intellectual functioning and academic ability. The results show that a subset of MOGAD patients experience clinically significant sequalae in intellectual functioning and academic ability. The neuropsychological profile also differed between children with and without MOG-ab positivity, with seronegative patients more likely to show a clinically relevant difficulties at the individual patient level. Whilst no differences existed at the group-level; the current study demonstrates the relative additional risk of intellectual/academic difficulty associated with MOG-ab seronegativity. This research further supports the growing perspective that MOG-positivity confers a more favourable neuropsychological outlook than is the case for their seronegative counterparts. This broadening consensus offers reassurance for clinicians, families, and patients.

## 1 Introduction

Paediatric acquired demyelinating syndromes (pADS) affect 9.83 per million UK children (1yr–15yr 11m) per year [1]. pADS specifically attack white matter fibres within the central nervous system (CNS), during a period when the developmental refinements of this neural ‘wiring’ impact on learning and neurodevelopment (i.e. [2–4]). This likely explains why there is significant evidence to suggest that acquired demyelination of the CNS in children impacts their developing cognitive abilities [5]. Subsequently, these children face three and a half times greater risk of mental health comorbidity [6], are less likely to attend university, and have greater reliance on disability benefits with lower employment earnings [6,7]. Understanding the neuropsychological profile of these children will help us to understand the specific support needed to attempt to ameliorate these poor, long-term functional outcomes.

Current research suggests that neuropsychological phenotypes in paediatric ADS are heterogeneous, mostly due to complexity of disease. Multiple disease processes and factors differentially affect the still developing CNS and therefore outcomes, such as age of disease onset, disease burden (e.g., lesion presence, number, and location), disease duration and disease course (e.g., relapsing verses monophasic disease and number of relapses). See Tan et al. [5] for an extensive review. Understanding risk factors for poor outcome will guide clinical management of these children.

One clinical phenotype which could differentially impact outcomes is the presence of autoantibodies to myelin oligodendrocyte glycoprotein (MOG). MOG-ab associated disease (MOGAD) is an autoimmune demyelinating syndrome associated with antibodies against MOG with a higher incidence in children, with variable phenotypes at presentation (ADEM, ON, TM, neuromyelitis Optica spectrum disorders (NMOSD) and/or encephalitis) but research is still full characterising this condition [8–10]. Presence of these antibodies have specific consequences for relapse risk and therefore clinical management [11–14]. Importantly, the clinical phenotype of pADS presenting with MOG-ab is strongly mediated by age [15] and thus, research focussing explicitly on pediatric cohorts is of paramount importance.

Recent research has investigated long-term cognitive impairment and disability as a potential sequalae of pediatric-onset ADS patients who test positive for MOG-Ab, termed MOGAD. Cognitive comorbidities are thought to be common but are poorly delineated due to limited studies [16]. Clinician-reported cognitive impairment (assessed via survey [17] or neurological examination [18]) in MOGAD patients has been estimated between 4 and 12 % of cases, even at long-term (approx. 5 years) follow-up [18]. In a retrospective cohort study of MOGAD patients, the Expanded Disability Status Scale (EDSS) identified only one paediatric patient (9.1 % of overall pediatric cohort) having residual cognitive deficit [19]. However, subjective ratings of what constitutes ‘impaired’ functioning can be highly variable between clinicians [20], and even quantitative tools such as the EDSS are more representative of the physical disabilities as opposed to cognitive difficulties [21]. These may therefore inaccurately estimate, or underestimate difficulties, compared to studies which use standardised neuropsychological assessment tools. A retrospective case series, using standardised tools, suggested that the majority of MOGAD patients (approx. 8 months follow-up) had intact cognitive functioning (71 %) whilst the remaining had difficulties explainable by confounding factors such as premorbid learning difficulties [22]. Importantly however, these neuropsychological tools identified difficulties not captured by the EDSS [22]. Varying definition and methodology for assessing cognitive functioning results in poorly delineated cognitive comorbidity [16]. Further research with standardised testing tools is necessary to further elucidate whether MOG-ab is associated with poor cognitive phenotypes.

There is limited research to highlight whether the presence of MOG lgG (i.e., MOG positivity) is a specific risk factor for poorer cognitive outcomes in these cases, specifically compared to seronegative ADS groups. Whilst it is acknowledged that POMS and MOGAD represent two distinct demyelinating syndromes [23], there is evidence to suggest that the mere presence of autoantibodies can mediate neuropsychological outcomes in children. A recent study of autoimmune encephalitis found similar patterns between NMDA-r encephalitis, and probable or seronegative encephalitis [24], with greater difficulties seen in the seronegative cases.

Early indications suggest a similar or less severe profile of difficulties in MOGAD compared to other pediatric-onset ADS [17,22,25]. A recent study compared neuropsychological profiles between relapsing pADS cases, specifically MOGAD, pediatric-onset Multiple Sclerosis (POMS) and healthy controls [25] at long-term (5–13year) follow-up. Both patient groups (MOGAD/POMS) demonstrated difficulties (compared to healthy controls), however difficulties in the MOGAD patients were found specifically in complex reasoning, whilst for POMS patients, a broader reduction in performance was found across multiple domains of executive function, episodic memory, and complex cognition, as well as slower reaction times. This supports a picture of a more limited profile of difficulties seen in MOG patients [25] compared to other pADS cases.

For these paediatric patients, a substantial portion of their time is spent on formative education, and thus neuropsychological assessment must also take into consideration academic difficulties that could affect patients’ participation in school. A recent, multi-institutional, prospective UK study reported levels of physician-reported difficulties across multiple domains, including educational difficulties, in children with ADS [26]. Of the children diagnosed with relapsing MOGAD (n = 12), 33 % were thought to have educational difficulties, compared to 17 % in confirmed seronegative relapsing ADS cases (despite the group having a median EDSS of 1). Previous research, using parent-report, estimates levels of school support in a large sample of paediatric MOGAD at around 26 % at long-term (~4.5 years) follow-up [27]. Interestingly, no differences were found between patients with relapsing versus monophasic disease, indicating these difficulties can present even after a single event. Specifically, support consisted of being in receipt of academic accommodations (e.g. additional school support or specialist placement). Rates of school performance difficulties were comparable (approx. 20 %) in a study of a paediatric cohort [12]. Whilst ecologically valid [28], these parent-reports of school support are dependent on several factors beyond academic difficulty (e.g., local school resourcing, level of parental/doctor advocacy). This therefore represents a high-bar for the definition of academic difficulty and, given support may take time to agree and put in place in education settings, may display some lag. Overall, these studies may therefore underestimate actual difficulties. This study will specifically evaluate education difficulties with an age-appropriate, standardised assessment of academic achievement, allowing an estimate of specific domains of difficulty, more independent of the socio-economic factors outlined above.

Work to date has mostly focussed relapsing MOGAD, with little research on cognitive difficulties in monophasic MOG lgG positive cases. Tan and colleagues (2021) included both monophasic and relapsing patients but, due to limited sample sizes, and relatively good outcomes across the whole cohort, conclusions regarding differences due to disease course are limited. Deiva and colleagues (2020) found no differences in number of children receiving school support between monophasic and relapsing disease course. In monophasic ADS cases, focus is on acute treatment and resolution of neurological symptoms, with the view that, upon resolution and baring relapse, that the child be considered well. However, recent research has challenged this, with even a single demyelinating event sufficient for poor outcomes, and even atypical neurodevelopment, with children failing to recover the normative developmental trajectory after recovery [29–31]. The current study focuses on a heterogenous sample of MOG-ab positive and negative ADS cases, including both monophasic and multiphasic illness.

There is not sufficient research to reach a current consensus of the profile or trajectory of intellectual functioning and educational difficulties in MOGAD. Highlighting the specific profile seen in these children, in comparison to similar cases who do not test positive for this autoantibody, may inform us about the specific support needed to support these patients, or even brain networks and processes which are being specifically impaired in MOGAD.

The current study assesses children diagnosed with MOGAD or pADS who are seronegative for MOG-ab, using standardised assessments of both cognition and academic ability, to answer the following research questions.

- Do MOGAD patients experience neurocognitive sequalae in terms of intellectual functioning and academic ability?
- Following a diagnosis of ADS, does the neurocognitive profile differ between children with and without MOG-ab positivity?

## 2 Methods

### 2.2 Ethics

The current project received ethical approval from Aston University and NHS Research Ethics Committees (reference #18/LO/0990; #IRAS 233424) to recruit patients, and Aston University College of Health and Life Sciences Ethics committee for recruitment of healthy controls (reference #HLS21011). In all instances, informed consent was obtained from parents or guardians by the research team to take part in the research study. Verbal or written assent was also sought from child participants.

### 2.2 Participants

pADS patients were retrospectively recruited from the Birmingham Children’s Hospital (part of the Birmingham Women’s and Childrens NHS Foundation Trust). Patient families were initially approached by clinicians, followed by researcher contact for those families who were interested in taking part. pADS patients were eligible if diagnosed with an acquired demyelinating disease (e.g., Acute Disseminated Encephalomyelitis (ADEM), Optic Neuritis (ON), Multiple Sclerosis (MS), MOGAD etc.), and had undergone a clinical MRI scan at least 18 months prior to recruitment. MOG is prevalent in both ADS, but also in paediatric encephalitic phenotypes (MOG-Ab found to be most common neuronal autoantibody in prospective observational cohort of encephalitis [32]). Cortical encephalitic phenotypes of MOGAD have also been described in children [33]. Therefore, children with MOG-ab positive (MOG-antibody positive) encephalitis were also recruited as part of the MOGAD group for the current study.

Additional inclusion criteria included i) aged between 6 and 16 years old at time of recruitment, ii) sufficient English-language ability to not require an interpreter, and iii) no contraindications for MRI scanning.

Healthy controls were recruited from the local community through social media advertisement and local outreach events within the Birmingham area. Healthy controls were included only if no known (or suspected) cerebral abnormality, with no neurodevelopmental diagnosis (e.g., Autism, ADHD etc.). Despite the existence of published norms for the standardised testing tools described here, we recruited a control cohort to capture potential deviations from these published norms in the context of the local (geographic) population under investigation. Given the limited sample sizes, we did not attempt to conduct matched recruitment of the healthy control sample, beyond recruiting from the same geographic area (both the research centre and the children’s hospital are within the same central Birmingham location).

Participants were categorised into three groups: a) MOGAD, b) pADS - MOG-ab negative and c) healthy controls. pADS patients were assigned to either the MOGAD or MOG-ab negative group, based upon whether a positive MOG-ab assay result was found in their medical notes.

Clinical and demographic data was collected from participants (using study-specific demographics questionnaire) and clinical variables collected through review of medical records by clinician-researchers.

### 2.3 Measures

#### 2.3.1 Intellectual functioning assessment

Assessment was conducted by trained researchers, supervised by the principal investigator (AW) who has significant clinical experience of the tools. Assessments were conducted as part of a wider research protocol, and children were given the opportunity to take breaks at any point.

Intellectual functioning was assessed using the Weschler Intelligence Scale for Children – Fifth UK Edition (WISC-V, [34]), a standardised and normed (to UK sample) intelligence assessment valid for children aged between 6 and 16years old. Full assessment was conducted to calculate five primary index scores of the WISC-V (Verbal Comprehension, Visual Spatial, Fluid Reasoning, Working Memory & Processing Speed) and the full-scale IQ (FSIQ) score. All scores were age-scaled using the WISC-V UK normative data (M(SD) = 100(15)).

#### 2.3.2 Academic assessment

Academic abilities were assessed using the Wechsler Individual Achievement Test - Third UK Edition (WIAT-III, [35]), a standardised assessment of reading, language, and numerical attainment. The test is valid and normed (to a UK sample) for children and young-adults between 4 and 25yrs old. Of the 8 available composite scores, subtests were conducted that allowed measurement of four specific composite scores; Total Reading, Basic Reading, Reading Comprehension and Fluency and Mathematics (see Table 1). All scores were age-scaled using the WIAT-III UK normative data (M(SD) = 100(15)). Healthy control participants did not complete the WIAT due to being recruited under a different testing protocol.

Supplementary, parent-reported information was gained from demographic questionnaire and responses to the academic-functioning rating scales on the Child Behavior Checklist (CBCL, [36]). The study-specific demographic question asked parents “Does your child receive any additional support at school?” with a yes/no answer, followed by a free form text box to provide details. The academic-functioning rating scales of the CBCL included two questions on school-support; “Does your child receive special education or remedial services or attend a special class or special school?” and “Has your child repeated any grades?”

### 2.4 Statistical analysis

One-way ANOVA and T-tests were used to assess whether groups (MOGAD, pADS (MOG-ab negative), HCs) differed on demographic and disease variables. Where assumptions of normality were violated, Wilcox’s robust methods (calculated using median values) were used [37, 38]. The same approaches were used to assess whether groups differed on index/composite scores from the WISC-V and WIAT-III.

Previous research in paediatric-onset ADS has highlighted that whilst mean performance on neuropsychological assessment may fall into the average range, a subset of patients may still experience significant difficulties [5,39]. Therefore, individual-level analyses were conducted to investigate across how many index scores (across the 6 index scores in the WISC-V and 3 from the WIAT) performance fell below reference scores of clinical-concern (1SD or more below the test mean, Scaled score between 70 and 85) or clinical impairment (2SD below test mean, Scaled score below 70). For each participant, the frequency of “clinically concerning” or “clinically impaired” scores are reported.

A neuropsychological impairment (NPI) rule [40] was also used to identify individuals as demonstrating reduced performance versus those who did not using a cut-off based upon the number of cognitive indicators (Index/Composite scores) falling below the chosen performance (in this case >1SD below test mean). We used a range of cut-offs including one, two and three domains being below performance threshold to indicate ‘poor’ outcome. Number of individuals with versus without a poor outcome for each of the two patient groups (MOGAD vs pADS (MOG-ab negative)) were compared using Fisher’s Exact Test.

Exploratory analyses (using Pearson correlations and ANOVA for continuous and categorical data respectively) investigated whether disease factors (disease duration (years), age at onset (years.), disease course (mono versus multiphasic), ADEM-like presentation) were associated with outcome measures. For these analyses, patients (MOGAD vs pADS (MOG-ab negative)) were treated as a single cohort.

All analyses were conducted using RStudio ([41]0.03.0 + 386).

## 3 Results

### 3.1 Patient and healthy control demographics

Twenty-one pADS patients were recruited, n = 10 who tested positive for MOG-ab and were assigned to the MOGAD group and n = 10 who did not (pADS (MOG-ab negative)). Eleven healthy control children (HC) were also recruited. Demographics characteristics of each group can be seen below (see Table 2). For the pADS patients, minimum time since disease onset was 1.77 years prior to recruitment, whilst the maximum was 11.18 years prior. Mean age of onset was approximately 7 years old for both subgroups. Those pADS patients with and without the MOG-ab did not significantly differ in terms of disease duration, age at onset or disease course (proportion of monophasic vs. multiphasic). Diagnoses for each of the patient groups is reported in Table 2. One HC participant was removed from subsequent analysis due to an FSIQ score falling more than 2SD below test mean.

### 3.2 Group-level comparisons

**Fig. 1:**
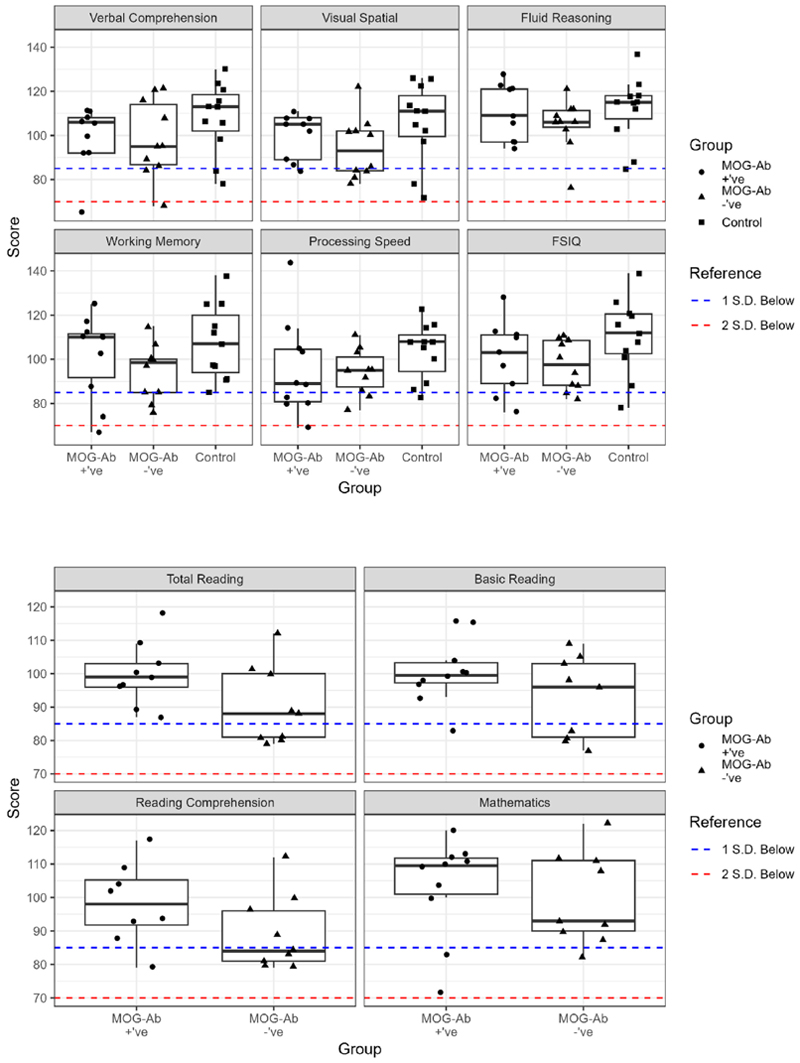
Boxplots to visualise distribution of scores on a) the 6 WISC-V index scores and b) the 4 WIAT-III composite scores, across the three groups. *Median scores are plotted, whilst the lower and upper hinges correspond to the first and third quartiles (the* 25th *and* 75th *percentiles). Upper/lower whiskers represent largest/smallest value within 1.5 times the IQR above/below the hinge*. Reference lines represent 1 and 2 SD below mean test performance (M(SD) = 100(15)).

**Fig. 2:**
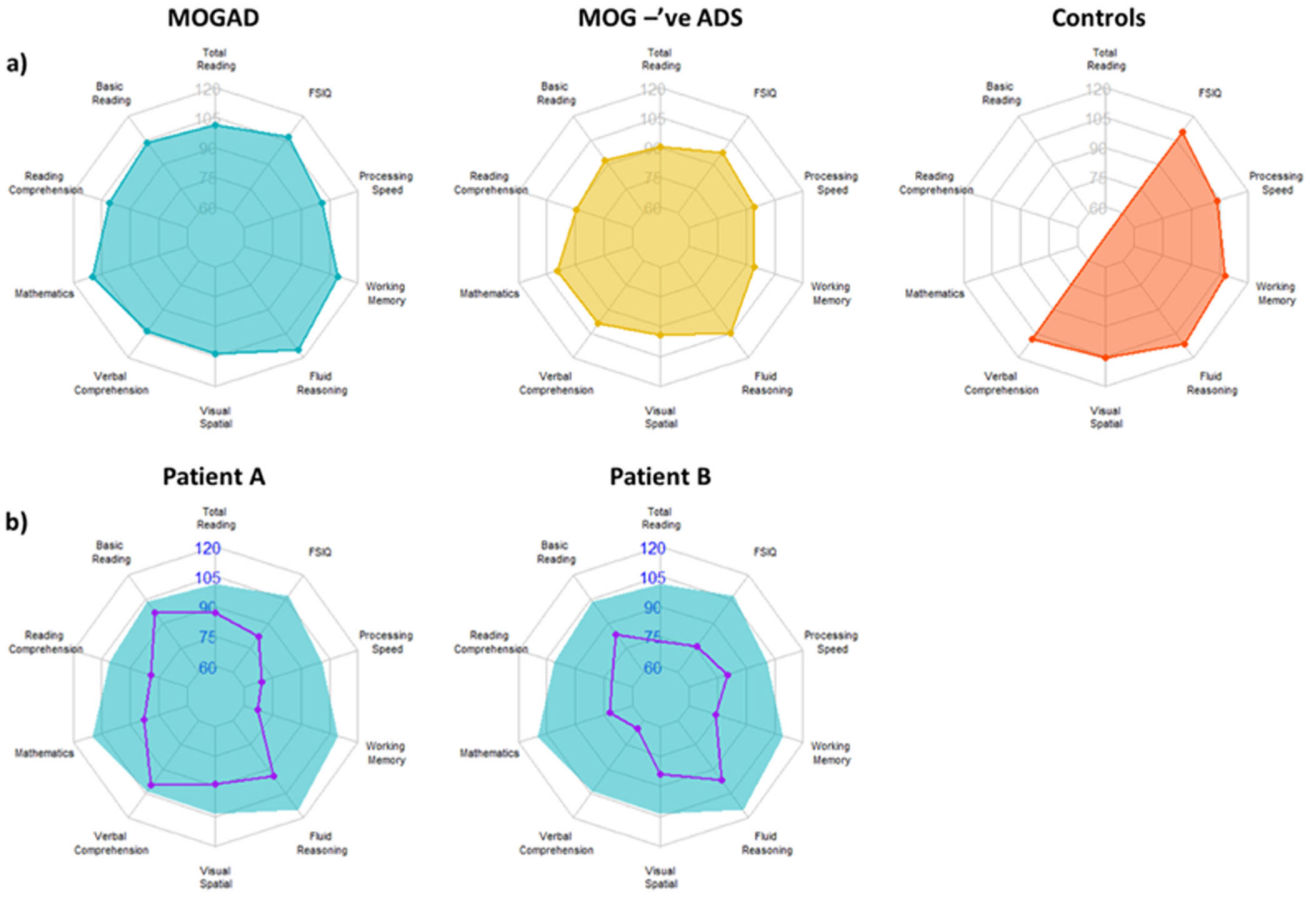
a) Radar plots representing average performance on each domain of functioning for MOGAD patients, seronegative patients (MOG-ab –’ve pADS) and controls. The MOGAD group represents the average with the two patients with greater than two domains of clinically concerning scores (at 1SD) being removed. Whilst not significantly different, qualitative viewing of the data is suggestive towards a greater level of difficulties in seronegative cases. b) Individual profiles for the two patients with a greater number of clinically-concerning/impaired domains, is displayed with reference to the average MOGAD profile, showing the multiple and broad difficulties seen in these patients.

Mean performance for index/composite scores collected using the WISC-V/WIAT-III for each group is reported in Table 3. No significant effect of group on outcome scores was found, across all scores (p > 0.05 (uncorrected) for all comparisons). Boxplots of scores showing individual-level performance can be seen in Figs. 1 and 2.

In an exploratory analysis, one-way T-test against test norm/mean of M = 100 was performed for PSI, as the only index score where median performance for the MOGAD group fell below the mean of both the MOG-ab negative and HC groups (see Fig. 1). A directional alternative hypothesis, that group mean was lower than test mean, was adopted. The mean of the MOGAD group was not significantly below test norm (t (9) = -0.639, p = 0.269), except when the one highly performing individual (>2.5SD above test norm) was removed from the data (t(8) = -2.354, p = 0.038). For completeness, neither the mean for the MOG-ab –’ve or HC groups were significantly below the test mean (t(9) = -1.765, p= 0.557, & t(10) = 0.936, p = 0.815 respectively). Exploratory analyses were only conducted on scores where the MOG-ab positive group were below all other groups given this was the patient group of interest for the current study.

### 3.3 Individual-level performance

Table 4 shows the frequency of participants performing 1SD below the test mean for each of the index/composite scores, for each group. Few participants in any group met the conservative impairment threshold of 2SD below test mean (see supplementary table S1). For the remaining comparisons we describe results pertaining to performance which is 1SD below test-mean as an index of clinical-concern (see Table 5).

In the MOGAD group, most common difficulty in intellectual functioning was seen in Processing Speed Index (PSI; 40 %), with only three cognitive domains achieving threshold for clinical-concern more than one child (Processing Speed, Working Memory and IQ). In terms of academic ability, mathematics was most commonly affected in the MOGAD group (20 %). When measuring performance in both intellectual and academic domains, four children had difficulties in terms of performance on one or more domain for the MOGAD patients (40 %) compared to nine children in the pADS (MOG-ab negative) group (90 %). It is important to note that four HC children (36 %) exhibited clinically concerning scores in 1 or more domains of functioning. Table 4 highlights these results.

Of the two MOGAD cases with greater than two domains of clinically concerning scores (at 1SD), they had multiple and broad difficulties (see Fig. 2). The first patient (Patient A) had difficulties in 50 % of domains evaluated, diagnosed at approximately eight years old, and was being tested ~2.5 years post-diagnosis. Their disease phenotype was MOG-Ab positive, multiphasic ADEM. The second (Patient B) had clinically concerning scores in 87.5 % of domains evaluated, diagnosed at ~5 years old and was being followed-up at two years post-diagnosis. Their disease phenotype was a monophasic MOG-Ab positive encephalitis. It is important to note that these two patients were the only MOGAD patients with any clinical impairment at a cutoff of 2SD (20 % and 12.5 % (respectively) domains impaired at 2SD below test mean).

In the pADS MOG-ab negative group, most common intellectual difficulty was seen in Visual Spatial Index (VSI) and Working Memory Index (WMI), both 40 % of children, with five cognitive domains rated as clinically concerning for more than one child (frequency of clinically concerning scores ranged from 10 to 40 % on WISC-V index scores). Reading comprehension was most commonly affected academic domain for the pADS MOG-ab negative group (55.6 %). In the pADS MOG-ab negative group, all composite scores of academic abilities were clinically-concerning for one or more children (from 11.1 to 55.6 %), whilst a lower frequency of clinically concerning scores was observed in the pADS MOG-ab negative group (3 out of 4 domains with one or more children demonstrating clinically concerning scores, from 10 to 20 % of the cohort had difficulties across these academic domains).

Given the lack of specificity of individual index scores in discriminating between groups, a further approach assessing performance more broadly across multiple domains was considered. The NPI rule was used to identify individuals as demonstrating a reduced performance versus those who did not’ using a cut-off based upon the number of domains falling >1SD below test mean. This was to test the hypothesis of a ‘broad’ phenotype of difficulties seen in some demyelinating disease, irrespective of domain. Significantly more individuals identified by the NPI rule were found in the MOG-ab negative group, compared to the MOGAD group when the threshold for difficulties was set at 1 or more test scores falling >1SD below test mean (X^2^ (1) = 5.49, p = 0.019) or 2 or more test scores falling below 1SD (X^2^ (1) = 7.20, p = 0.007).

When the NPI rule was set at *two* or more domains rated as clinically-concerning at 1SD below test mean (a-priori selected as the most discriminate threshold between groups), “impaired” versus “non-impaired” individuals did not differ in terms of other factors, across patient (age and sex), or disease characteristics (disease duration, disease onset, disease course). This suggests that factors other than MOG-ab status do not better explain this difference in number of individuals identified by the NPI rule (in this dataset).

### 3.4 Effect of disease factors

Exploratory relationships between disease factors and WISC/WIAT index scores were assessed using the whole cohort (n = 20) of patients (grouping both MOGAD & pADS (MOG-ab negative) cases). No significant correlations (uncorrected for multiple comparisons) were found between disease factors (disease duration (years), age at onset (years)) and any outcome measures. Using ANOVA, differences were neither found in outcomes due to disease course (mono-versus multiphasic) or ADEM-like presentation (binary yes/no). Given that these analyses were conducted independent of testing for the affect of MOG antibody status, these results indicate that no other disease phenotypes were associated with performance at the group level.

### 3.5 Parent-report of school support

Families of nine MOGAD patients and 10 MOG-ab negative pADS patients reported on whether patients received additional support at school. Of the antibody positive patients only one (14.3 %) reported having school support, specifically additional learning activities (e.g., additional classes). In the antibody negative patients, six (60 %) reported having additional school support, including adjustment to teaching (n = 4 e.g., small group teaching, additional breaks), having additional support staff (n = 3), and being held back a school year (n = 1).

## 4 Discussion

This study reports on assessment of intellectual functioning and academic performance for a UK cohort of MOGAD patients. Using standardised neuropsychological assessments (specifically the WISC-V and the WIAT-III) the current findings suggest that, on average MOGAD patients, demonstrate normative intellectual and academic abilities, at least 18 months after diagnosis. Similar results were found when investigating individual-level performance (instead of group-level averages). In fact, the number of individuals identified as having ‘difficulties’ was greater in a group of seronegative PADS patients, compared to the MOGAD patients. Overall, these findings are reassuring for the longer-term sequalae in MOGAD.

However, there was still a small subset of MOGAD patients, with scores reaching threshold for ‘clinical concern’. When classifying patients as impaired/not impaired based on the number of affected index scores, with a threshold was set at one or two index scores, significantly greater number of individuals with cognitive difficulties was found in the antibody negative group (90 % and 80 %, respectively) compared to the MOG-ab group (40 % and 20 %). Rates of clinically concerning difficulties in the current MOGAD group were therefore in the upper range of what has been estimated by previous studies estimating cognitive difficulties using clinician reports. In a questionnaire survey based upon retrospective paediatric-onset ADS cases in Japan, clinicians reported specific cognitive impairment in approximately 4 % of MOG lgG cases, similar rates to that of seronegative groups [17]. Cognitive disability, assessed via neurological examination in a lifespan cohort (56 % of which were paediatric cases) at around 5yr follow-up, was identified in 12 % of relapsing MOG cases [18].

Yet, in the current MOGAD group, it is important to reiterate that both reduced intellectual functioning and academic performance was less common overall compared to the seronegative group. Overall, this suggests that MOGAD may result in a narrower profile of difficulties when affected, and a lower likelihood of clinically concerning scores. This echoes previous findings of a limited profile of difficulties seen in MOGAD [25] or even largely intact neuropsychology profiles for these patients [22].

In Fabri et al [25], patients with MOGAD demonstrated specific difficulties in complex reasoning tasks, and slower response times compared to controls, whilst POMS patients showed broader difficulties across multiple domains of executive function, episodic memory, and complex cognition, as well as slower reaction times. These domains are somewhat similar to those found in our cohort when investigating the number of patients reaching clinically concerning thresholds for each index score. For the antibody negative patients, working memory and visual spatial skills were most frequently affected (40 %), whilst for the MOGAD patients, the most commonly impacted index score was processing speed with 40 % of cases demonstrating clinically-concerning scores. It is important to note that, when removing a high performing outlier in the MOGAD group, mean performance for processing speed was significantly below test norm, and thus this may be indicative of an antibody specific deficit – however, these analyses were exploratory and should be a target of future investigations.

However, it is important to note that the cognitive difficulties seen in Fabri and colleagues’ (2022) study were at the group level, with differences in performance seen between MOGAD, POMS and HC groups, whereas we focussed on the subset of patients with clinically concerning difficulties. Our lack of significant differences in distribution of test scores between HC, and antibody positive and negative groups may be due to the lack of statistical power and smaller sample size in the current study. Whilst the MOGAD group in the Fabri and colleagues’ study (2022) was only slightly larger than the MOG group in the current study (n = 12 vs n = 10), the previous study used a more homogenous sample, focussing only on relapsing patients. The primarily monophasic sample used in the current study may indicate that monophasic, seronegative demyelination is more likely to experience neurocognitive sequalae compared to the MOG-ab positive cases. Future research should attempt to include both monophasic and relapsing cases to identify whether MOG-related difficulties may exist outside of relapse-associated worsening (in relapsing cases) but also in monophasic cases.

Children spend a substantial portion of their time on formative education, and thus assessment must also take into consideration academic difficulties that could affect patients’ participation in school. Using the WIAT-III to assess academic achievement in domains of reading and mathematics, again no differences were found between groups. For the MOG-ab positive group, difficulties were infrequent with reading comprehension most common (14.3 %). However, in the MOG-ab negative group, difficulties were more common, with all reading composite scores (Total/Basic Reading and Reading Comprehension) having a frequency of difficulties at 44%–55 % of the group. The frequency of academic difficulties for the MOGAD group was lower than estimated in previous research using more qualitative approaches. Across a cohort of paediatric ADS cases, all of whom tested positive for MOG lgG, approximately 26 % presented with academic difficulties (defined as being in receipt of academic accommodations such as requiring additional school support, repetition of school grades or special education placement) at an average follow-up of 4.5 years [27]. Most frequent difficulties were seen in younger (<10years old) patients, and those initially presenting with an ADEM-like phenotype or deep grey-matter lesions [27]. Rates of school performance difficulties were comparable (approx. 20 %) in a study of a paediatric cohort [12]. Using parental self-report pertaining to school support, the current study found higher rates of support for the seronegative group (6 children), whereas in the MOGAD group only one child received additional support. Limited sample sizes precluded investigation in the current study, but future research should investigate associations between these and cognitive difficulties to investigate the potential role of cognitive impairment on wider functioning [12].

It is unclear if MOG-ab are truly pathogenic or a disease biomarker [9], however clinical phenotypes (e.g. age at onset etc) for MOGAD versus those seronegative for this antibody are significantly different [11]. These disease factors may in fact have an impact on neuropsychological outcomes [42], and therefore we investigated these factors in relation to our outcome measures. Due to the limited sample size, we combined the two patient groups into a whole pADS cohort. No significant relationships were found between measures of intellectual function and disease factors. Previous research has suggested that ADEM-phenotype for MOGAD is more likely to lead to poorer outcomes, including cognition [12,43,44], especially in younger children [11]. The current study did not replicate this finding, in line with Fabri and colleagues (2022) who showed that even non-ADEM presentations (e.g., ON, TM) can have an impact on domains of cognition. However, some of these differences observed in other studies may be due to the use of the EDSS, versus more formal standardised cognitive testing of intellectual function – the EDSS is heavily weighted towards motor rather cognitive difficulties [44]. A further disease factor to be investigated is the effect of treatment however, as treatment is individualised to patient phenotype and clinical scenario, a much larger sample would be needed to assess this.

MOGAD is thought to result in cognitive sequalae due to common grey matter involvement compared to other demyelinating disease [16]. Many children with MOG present with lesions on MRI, typically with a worse clinical and radiological presentation than non-antibody counterparts [9,45]. Despite this, many patients show partial or full resolution of MRI abnormalities over time [32,45–49]. This resolution of lesions could be the clinical phenotype which differentiates the less severe neurocognitive profile apparent in MOG-ab positive cases of pADS. Essentially, they typically recover well, with low EDSS and disability in monophasic disease [10], with our results suggesting better neuropsychological outcomes as well. These factors may also explain why, in studies of relapsing MOGAD (e.g. Fabri et al. [25]), slightly worse cognitive phenotypes are seen for these patients than in the current study involving monophasic patients, with relapsing disease likely to result in new lesions [50]. Given this, and recent evidence of ‘silent’ new MRI lesions even in monophasic MOGAD patients [51], future research should integrate MRI data with these cognitive outcomes to try and explain cases in the MOG group who do experience difficulties in the longer term. Longer-term assessment will also allow us to assess these outcomes in relation to dynamic nature of these lesions, and the potential for relapse in our currently monophasic patients.

## 5 Limitations

An important consideration for the current results is the definition of clinically concerning scores. Test scores alone do not define or necessarily capture cognitive impairment, and it is important to consider the combination of factors (including test-scores that deviate from normative expectations) alongside functional capacity [20]. The test scores identified as ‘clinically-concerning’ for this patient group used the threshold of >1SD below the mean normative test score, which equates to ‘Low Average’ functioning and below [20,34,35]. In the general population, this may be considered an over-zealous definition of cognitive difficulties however, given the medical history of these patients we selected this threshold as a meaningful index of ‘concern’ which may instigate further investigation if presenting to a neuropsychology clinic. The parent-reported information regarding school support (as a proxy for functional capacity) echoed our ‘findings in that the MOG-ab negative pADS patients had a greater frequency of support offered/required than the MOGAD patients.

Significant limitation amongst this and previous research is the focus upon those children aged six years old and above. This is likely pragmatic in terms of recruitment to research and assessment of children. Given the double hazard model [52,53], suggests that the combination of more severe brain insult, plus younger age at injury leads to poorer outcomes than either factor alone, it will be important to also study these younger patients with age appropriate and comparable neuropsychological assessment tools. It is important to also note that no new antibody testing/assays were used to validate current antibody status for any patients in the current study – antibody presence (positive/negative) was entirely based upon thorough review of the medical records.

Larger studies will be needed to further investigate the role of interacting factors which may influence outcomes as a function of antibody status. For instance, MOGAD does not show the female predominance seen in other pediatric acquired demyelinating syndromes such as MS and neuromyelitis optica spectrum disorders [54]. There is however, evidence to suggest that cognitive outcomes across demyelinating disease in children, differ as a function of sex. In paediatric ADEM, there is greater risk of neurological poor outcome (including intellectual difficulties) for males [55]. Similar trends are seen in paediatric-onset multiple sclerosis; males are more likely to be cognitively impaired [56], experience further decline in cognitive processing speed [57] and show diminished cognitive functioning even at 2–5yrs post-onset [58]. Given the likely interaction between sex differences in phenotype and outcome, this is an area of explicit interest for future research, however was beyond the scope of the sample size in the current investigation.

One important factor to be considered in future cognitive research for this population is that the full impact of these diseases may not be detected early, and may in fact become more apparent over time when typical developmental milestones occur [11]. Current research has focussed on the medium-term outcomes (18m onwards), but it will be important to consider very long-term outcomes in this group also. This is particularly important to inform school transition from primary to secondary education when the demands on children’s cognitive skills turn more to executive functioning and deficits may emerge.

## 6 Conclusion

The current study assessed children diagnosed with pADS who tested positive or negative for MOG-ab using standardised assessments of both intellectual functioning and academic performance. Specifically, we found that a small subset of MOGAD patients do experience neurocognitive sequalae, however this is less likely than for seronegative pADS cases, with a much narrower distribution of domains affected. This was reiterated in parent-report data showing more seronegative patients were receiving school-support than those testing positive for the antibody.

Future efforts should be made towards collaborative consortium studies that facilitate recruitment of larger yet diverse samples to further elucidate the cognitive profile of these patients, using standardised neuropsychological tools. This will allow us to disentangle the likely complex picture of specific risk and resilience factors related to these difficulties.

Despite being a newly detected clinical phenotype of an already rare disease, significant advances have been made to elucidate the presentation, outcome and sequalae for these children. Our research further supports the growing perspective that these cases have a more favourable neuropsychological outlook than seronegative counterparts. This broadening consensus in current research should be reassuring for clinicians, families, and patients. Further research in this area, to replicate this finding and investigate additional clinical risk factors influencing this difference, will lead to greater benefit in counselling patients and parents.

## Supplementary Material

Supplementary data to this article can be found online at https://doi.org/10.1016/j.ejpn.2024.09.001.

Supplementary data

## Figures and Tables

**Table 1:** Description of Index and Composite scores from the WISC-V and WIAT-III.

Assessment Tool	Index/Composite Score	Assesses?
WISC-V	Verbal Comprehension (VCI)	The ability to explain things using language.
	Visual Spatial (VSI)	The ability to understand visual details, the visual spatial relationships in construction problems and to integrate visual and motor skills.
	Fluid Reasoning (FRI)	The ability to resolve mental operations without using language.
	Working Memory (WMI)	The ability to attend to and hold information for a short period of time while thinking about it.
	Processing Speed (PSI)	The pace at which the child responds to information and tasks
	Full Scale IQ (FSIQ)	General intelligence based on a range of thinking tasks described above.
WIAT-III	Total Reading	Skills at reading comprehension, decoding words and nonwords, and oral reading fluency
	Basic Reading	The ability to read real and made-up words
	Reading Comprehension and Fluency	The accuracy with reading, and ability to answer questions based on those readings
	Mathematics	The ability to solve written math problems, identify geometric shapes, solve multi-step problems, and identify numerical patterns.

**Table 2:** Participant demographics.

	MOGAD	pADS (MOG-ab -’ve)	HCs	Difference
**Sample Size**	n = 10	n = 10	n = 11	
**Age (yrs.)**				
(Mean(SD))	10.41 (2.99)	12.10 (3.06)	11.44	F(2) = 1.76, p = 0.039^†^
(Min – Max)	6.87–15.63	7.53–15.62	(3.07) 6.19-15.78	
**Sex**				
(M:F)	3:7	3:7	8:3	p = 0.072^a^
**Disease Duration (yrs.)**				
(Mean(SD))	3. 46 (1.58)	4.93 (2.97)	NA	F(1) = 1.914, p = 0.183
(Min – Max)	1.1.77–7.20	2.09–11.18	NA	
**Age at Onset (yrs.)**				
(Mean(SD))	6.95 (2.93)	7.17 (4.20)	NA	F(1) = 0.019, p = 0.891
(Min – Max)	2.50–13.74	2.55–13.52	NA	
**Disease Course**				
(Relapsing: Monophasic)	4:6	2:8	NA	p = 0.628^a^
**Disease Phenotype at Testing**	MDEM (n = 1) ADEM (n = 1) AE (n = 2) Recurrent AE (n = 1) ON (n = 3) ADEM with ON (n = 2)	ADEM (n = 5) ON (n = 3) RRMS (n = 2)	NA	

† N.B. Robust ANOVA based upon median values, no post-hoc direct comparisons (Wilcox test) between groups were significant.

a Fisher’s Exact Test for Count Data Two-Sided Test, MDEM = Multiphasic

Disseminated Encephalomyelitis, ADEM = Acute Disseminated Encephalomyelitis, ON=Optic Neuritis, RRMS=Relapsing Remitting Multiple Sclerosis, AE = Autoimmune Encephalitis.

**Table 3:** Mean performance for index/composite scores collected using the WISC-V/WIAT-III for each group.

Index Score	Mean Performance (SD)*			Difference
	MOGAD	pADS (MOG-ab -’ve)	HCs	
WISC-V				
VCI	99 (15)	98 (18)	108 (16)	F(2) = 1.086, p = 0.353
VSI	100 (10)	94 (14)	106 (18)	F(2) = 1.182, p = 0.322
FRI	111(13)	105 (12)	112 (15)	F(2) = 0.787, p = 0.465
WMI	102 (19)	94 (13)	108 (17)	F(2) = 1.674, p = 0.206
PSI	96 (22)	94 (10)	104 (13)	F(2) = 1.120, p = 0.341
FSIQ	101 (17)	98 (11)	110 (17)	F(2) = 1.934, p = 0.164
WIAT				
Total Reading	100 (10)	90 (12)	NA	F(1) = 3.692, p = 0.073
Basic Reading	101 (10)	92 (12)	NA	F(1) = 2.617, p = 0.124
Reading Comp	98 (12)	89 (11)	NA	F(1) = 2.477, p = 0.136
Mathematics	103 (15)	100 (14)	NA	F(1) = 1.851, p = 0.056^†^

N.B. *to 0 decimal points,

† *Robust ANOVA based upon median values*.

**Table 4:** Frequency of individuals performing 1SD below test mean score (M(SD) = 100 (15)) for each domains tested using the WISC-V and WIAT-III.

Index Score	Frequency Per Group (%)		
	MOGAD	pADS (MOG-ab –’ve)	HCs
WISC-V			
VCI	1/9 (11.1 %)	2/10 (20 %)	2/11 (18.2 %)
VSI	1/9 (11.1 %)	4/10 (40 %)	2/11 (18.2 %)
FRI	0/9 (0 %)	1/10 (10 %)	1/11 (9.1 %)
WMI	2/10 (20 %)	4/10 (40 %)	1/11 (9.1 %)
PSI	4/10 (40 %)	2/10 (20 %)	1/11 (9.1 %)
FSIQ	2/9 (18.2 %)	2/10 (20 %)	1/11 (9.1 %)
WIAT			
Total Reading	0/9 (0 %)	4/9 (44.4 %)	NA
Basic Reading	1/10 (10 %)	4/9 (44.4 %)	NA
Reading Comp	1/8 (12.5 %)	5/9 (55.6 %)	NA
Mathematics	2/10 (20 %)	1/9 (11.1 %)	NA

*N.B. pADS* = *pediatric acquired demyelinating syndromes*, +*’ve* = *positive, -’ve* = *negative, WISC-V* = *Weschler Intelligence Scale for Children* – *5*th *UK Edition, VCI* = *Verbal Comprehension Index, VSI* = *Visual Spatial Index, FRI* = *Fluid Reasoning Index, WMI* = *Working memory Index, PSI* = *Processing Speed Index, FSIQ* = *Full Scale Intelligence Quotient, WIAT-III* = *Weschler Individual Achievement Test* – *3rd UK Edition, Reading Comp*. = *Reading comprehension and fluency*.

**Table 5:** Frequency and severity of individual clinically concerning scores per group, regardless of domain.

	MOGAD	pADS (MOG-ab -’ve)	HCs
Difficulties in 1 or more domains (n (%))	4 (40 %)	9 (90 %)	4 (36 %)
Difficulties in 2 or more domains (n (%))	2 (20 %)	8 (80 %)	2 (18.%)
Difficulties in 3 or more domains (n (%))	2 (20 %)	4 (40 %)	1 (9.1 %)
Median no. of domains* (range)	3(1–5)	2(1–7)	1.5 (1–4)

N.B. pADS = pediatric acquired demyelinating syndromes, +’ve = positive, -’ve = negative.

* = Only calculated for those individuals with clinically concerning scores in 1 or more domains.
