# Supplementary material for: Impact of autoantibodies against myelin oligodendrocyte glycoprotein in paediatric acquired demyelinating disease: Intellectual functioning and academic performance
Source: Eur J Paediatr Neurol. Author manuscript; Available in PMC 2025 Sep 4. (PMC7618078; doi:10.1016/j.ejpn.2024.09.001)

Supplemental Materials

| Table S1. Frequency of individuals performing 2SD below test mean score (M(SD)=100(15)) for domains tested using the WISC-V and WIAT-III | | | |
| --- | --- | --- | --- |
|  | Frequency Per Group (%) | | |
|  | pADS (MOG-ab +’ve) | pADS (MOG-ab -’ve) | HCs |
| WISC-V |  |  |  |
| VCI | 1 / 9 (11.1%) | 1 / 10 (10%) | 0 / 11 (0%) |
| VSI | 0 / 9 (0%) | 0 / 10 (0%) | 0 / 11 (0%) |
| FRI | 0 / 9 (0%) | 0 / 10 (0%) | 0 / 11 (0%) |
| WMI | 1 / 10 (10%) | 0 / 10 (0%) | 0 / 11 (0%) |
| PSI | 1 / 10 (10%) | 0 / 10 (0%) | 0 / 11 (0%) |
| FSIQ | 0 / 9 (0%) | 0 / 10 (0%) | 1 / 11 (0%) |
|  |  |  |  |
| WIAT |  |  |  |
| Total Reading | 0 / 9 (0%) | 0 / 9 (0%) | NA |
| Basic Reading | 0 / 10 (0%) | 0 / 9 (0%) | NA |
| Reading Comp. | 0 / 8 (0%) | 0 / 9 (0%) | NA |
| Mathematics | 0 / 10 (0%) | 0 / 9 (0%) | NA |
|  |  |  |  |
| *N.B. pADS = pediatric acquired demyelinating syndromes, +’ve = positive, -‘ve = negative, WISC-V = Weschler Intelligence Scale for Children – 5^th^ UK Edition, VCI = Verbal Comprehension Index, VSI = Visual Spatial Index, FRI = Fluid Reasoning Index, WMI = Working memory Index, PSI = Processing Speed Index, FSIQ = Full Scale Intelligence Quotient, WIAT-III = Weschler Individual Achievement Test – 3rd UK Edition, Reading Comp. = Reading comprehension and fluency*. | | | |

Figure S1. Graphs indicating no real difference in performance on cognitive (Top) or academic (Bottom) assessments between monophasic and multiphasic individuals, in the current cohort.


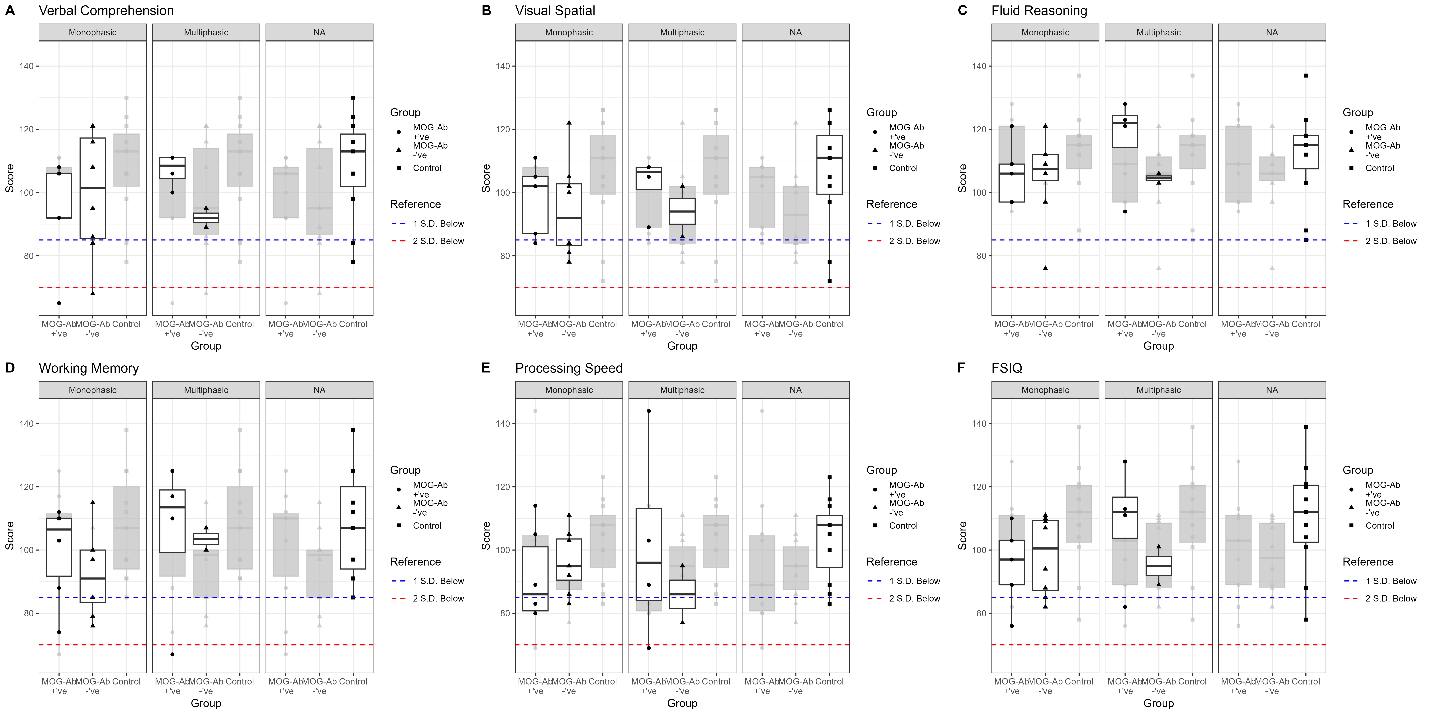


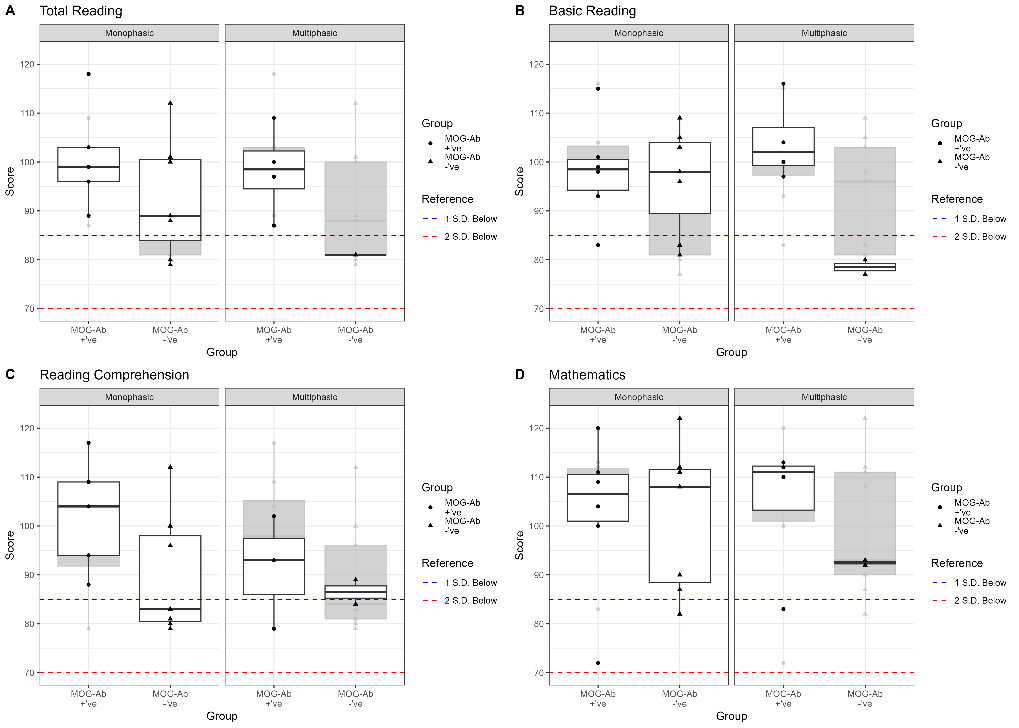

Supplement: Supplementary data [file EMS208259-supplement-Supplementary_data.docx]
